# Linking the Declarations of Helsinki and of Taipei: Critical Challenges of Future-Oriented Research Ethics

**DOI:** 10.3389/fphar.2020.579714

**Published:** 2020-10-29

**Authors:** Chieko Kurihara, Varvara Baroutsou, Sander Becker, Johan Brun, Brigitte Franke-Bray, Roberto Carlesi, Anthony Chan, Luis Francisco Collia, Peter Kleist, Luís Filipe Laranjeira, Kotone Matsuyama, Shehla Naseem, Johanna Schenk, Honorio Silva, Sandor Kerpel-Fronius, on behalf of Working Group on Ethics of the International Federation of Associations of Pharmaceutical Physicians and Pharmaceutical Medicine

**Affiliations:** ^1^ National Institutes for Quantum and Radiological Science and Technology, Chiba, Japan; ^2^ Independent Medical Consultant, and Consultant, Pharmaceutical Medicine, Athens, Greece; ^3^ Consultants in Pharmaceutical Medicine, Dover Heights, Australia; ^4^ LIF, Stockholm, Sweden; ^5^ Independent Consultant, Basel, Switzerland; ^6^ Independent Researcher, Bellagio, Italy; ^7^ Pfizer Biopharmaceuticals Group, Dublin, Ireland; ^8^ Craveri Pharma, Buenos Aires, Argentina; ^9^ Cantonal Ethics Committee, Zurich, Switzerland; ^10^ Eli Lilly & Co., Lisbon, Portugal; ^11^ Nippon Medical School, Tokyo, Japan; ^12^ Ferozsons Laboratories Ltd, Karachi, Pakistan; ^13^ PPH plus GmbH & Co. KG, Hochheim am Main, Germany; ^14^ IFAPP Academy, New York, NY, United States; ^15^ Semmelweis University, Budapest, Hungary

**Keywords:** research ethics, data science, medicines development, privacy protection, data sharing, Declaration of Helsinki, Declaration of Taipei

## Abstract

Expansion of data-driven research in the 21st century has posed challenges in the evolution of the international agreed framework of research ethics. The World Medical Association (WMA)’s Declaration of Helsinki (DoH) has provided ethical principles for medical research involving humans since 1964, with the last update in 2013. To complement the DoH, WMA issued the Declaration of Taipei (DoT) in 2016 to provide additional principles for health databases and biobanks. However, the ethical principles for secondary use of data or material obtained in research remain unclear. With such a perspective, the Working Group on Ethics (WGE) of the International Federation of Associations of Pharmaceutical Physicians and Pharmaceutical Medicine (IFAPP) suggests a closer scientific linkage in the DoH to the DoT focusing specifically on areas that will facilitate data-driven research, and to further strengthen the protection of research participants.

## 1 Introduction

Expanding interests in data-driven clinical science in the 21st century have posed some critical challenges in the recent evolution of research ethics. The International Council for Harmonization of Technical Requirements for Pharmaceuticals for Human Use (ICH) has endorsed renovation (ICH GCP Renovation, 2017) to facilitate utilization of reliable real-world data (RWD) for regulatory decision. This expands the usability of data derived from ordinary medical practice and research, as well as from health databases and biobanks. The World Medical Association (WMA) has since clarified some principles for these types of research but we believe it requires further clarity.

The WMA had established its paramount deontology of physicians to prioritize health and interests of a patient, as described in the Declaration of Geneva (WMA Declaration of Geneva, 1948) and the International Code of Medical Ethics (WMA ICoME, 1949), both issued in its second and third years of foundation. Since the first adopted version of the Declaration of Helsinki (DoH) in 1964 until the latest update in 2013 (WMA Declaration of Helsinki, 1964), the WMA has refined its core principle to prioritize rights and interests of the research subjects, ahead of scientific research goals. To implement this principle, with multiple DoH amendments, the WMA established an international agreed framework of ethics committee approval of research protocols, and the requirement of informed consent from research participants. It was its 5th amendment in 2000 that the scope of the DoH was expanded. Rather than limited to research involving individual humans, it would also cover research on identifiable human material or data. Since then, its scope has also been extended to include a framework of publication ethics: conflict of interest disclosure, publication of both positive and negative research results, and study registration in public databases.

Furthermore, reflecting decades of discussions concerning biobank developments in several countries, the 2008 amendment of the DoH added paragraph 25. It required researchers to justify the waiving of informed consent for research using identifiable human material or data, which may be obtained from biobanks or similar repositories, conditional upon ethics committee approval. In 2016 the WMA adopted the Declaration of Taipei, on Ethical Considerations regarding Health Databases and Biobanks (WMA Declaration of Taipei, 2016) (DoT), revised from its first version in 2002, to complement the DoH. It would now cover “the collection, storage and use of identifiable data and biological material beyond the individual care of patients”.

The scope of each of the two declarations is defined in both documents. However, it is not clear in the DoH how secondary or subsequent multiple use (we would describe these as “secondary use” hereafter) of data or material derived from “primary research” activity should be managed. From this point of view, we explored a way to clarify in the DoH to link with the DoT, as a part of our activities to promote ethical conduct of research (https://ifapp.org/working-groups/ethics-and-professionalism). The strengthened linkage to the DoT in the DoH, which is well-known worldwide as established principles for research involving humans, is required specifically for facilitating data-driven research while protection of research participants is maintained.

## 2 Crossroads of DoH and DoT

The DoT states that it provides “additional ethical principles” to the DoH. However, the DoH does not refer to the DoT. Therefore, it is a prerequisite to reference the DoT in any revisions of the DoH. Since the DoH deals with “research” and the DoT deals with “data/material collection”, the frameworks of these two types of activities have been separately considered. Therefore, investigators who are engaged in research without explicit intention of biobank/database development may not be aware of the governance framework defined in the DoT. Meanwhile, there is an increasing number of cases where the sponsors/investigators of the research or third party outside of the specific research later come to be interested in secondary use of data/material derived from it. For this reason, where there is a possibility of future secondary use of data/material collected in a research project, this research should be conducted adhering not only to the DoH but also to the DoT.

The essential requirements of the DoT which should be recognized by the research community are: 1) Items of information for obtaining “valid” consent when data/material are collected in a Health Database (HDB) or Biobank (BB) are defined including, e.g., the purpose of the HDB/BB; returning results including incidental findings; 2) Robust governance process of HDB and BB are defined including, e.g., documentation; traceability; arrangement of ownership change or closure; privacy protection and discrimination prevention; Material Transfer Agreement (MTA), all of which should be informed to the candidate donor of the data or material (WMA DoT, 2016; Dhai, 2016; WMA What we do).

## 3 Research in the Scope of the DoT and Valid Consent

Table 1 shows examples of HDBs and BBs and related examples of research and development (R&D) activities. Obviously, activities of development of HDBs, BBs and patient registries must adhere to the DoT.

**TABLE 1:** Examples of health databases and biobanks and examples of their utilization.

Types of health databases and biobanks with brief explanations	Related examples of expected R&D activities
**1. HDBs and BBs:** Project-based large-scale research resource development	Drug development lead candidate search
**2. Patient registry:** HDB development activity is sometimes associated with BB, focusing on one specific disease or intervention. Similar to cohort studies, but objectives are focused on research resource development rather than on simple prospective epidemiological research.	Rare disease drug development including lead candidate search. Alternative to control group of a clinical trial
**3. Real World Data (RWD):** RWD means data derived from ordinary medical practice. Recently, increasing number of repositories of de-identified data derived from RWD have been created.	New Drug Application (NDA) for new indication. Post-Marketing Surveillance (PMS) after expedited approval. Artificial Intelligence (AI) development
**4. Research involving human participants:** “Research” is not regarded as HDB or BB but there are increasing demands for secondary use from data/material obtained in the research.	Individual Participant Data (IPD) meta-analysis. Subgroup analysis of clinical trial results

Real World Data are being generated in the process of daily patient care, outside the scope of the DoH or DoT. However, recently, there has been an increasing number of activities for the development of HDBs to prepare anonymized or coded datasets for future secondary use. These activities are sometimes performed by commercial organizations under contract with a hospital/care organization, according to recently developed legal frameworks in various countries. The physicians’ ethical obligations to adhere to the DoT must be implemented in such processing of patient data.

Research involving human participants has not been typically regarded as HDBs or BBs. However, sometimes a researcher may only envision a possibility of future sharing of individual data/material with other researchers after the primary research has been completed but has not considered to inform the ethics committee nor the candidate participant. Such consideration should indeed be described in the study protocol and informed consent form (ICF), clarifying governance framework in accordance with the DoT, to be assessed by an ethics committee. Once the planned future sharing of data/material with the relevant governance framework is approved by an ethics committee, a candidate participant can then decide whether to accept or refuse this secondary use. This consent should be separately obtained from the consent to participate in the proposed primary research. The candidate’s decision whether to allow secondary use of data/material should not impact on possible participation in the primary research. Such consent does not mean traditional “broad consent” meaning “blanket consent” (Wendler, 2013) but “valid” consent as defined in the DoT.

Another aspect which needs clarification in the DoH is about the management of incidental findings (IFs). IFs are those identified during the research that are not primary objectives of the research project. Policy of reporting IFs is necessary part of valid consent in the DoT but it is not mentioned in the DoH. The right of an individual of taking option of knowing/not knowing the IFs should be assured in both DoH and DoT frameworks.

## 4 Individual Participant Data (IPD) Sharing and Trial Registration Requirement

On the premise of the above-mentioned governance framework both by the DoH and the DoT, we should consider the importance of “individual participant data (IPD) sharing” along with registration of a data sharing plan to a publicly available database, exploring the policies and statements issued from several international organizations, as shown in the Supplementary Material. Moreover, it is crucial that future IPD sharing is planned at the beginning of a research project, should be disclosed to, and approved by, the concerned ethics committee and then the volunteered participants.

“Data sharing plan” means the policy and planning of the way how the researcher can share IPD obtained in the research with other researchers for secondary analysis. The International Committee of Medical Journal Editors (ICMJE) stated in 2017 (Taichman et al., 2017) that responsible sharing of de-identified IPD of interventional clinical trials would be an “ethical obligation” and requires clinical trials enrolling participants on or after January 1, 2019 to include a data sharing plan in the trial registration. ICMJE allows researchers to register such a plan as “not available (we do not share our data)”, but each member journal editor may consider each plan during their editorial decision. Benefits and risks of data sharing are summarized in Table 2. Considering this situation, responsible IPD sharing should be recommended as an “ethical obligation” in the DoH.

**TABLE 2:** Benefits and risks of IPD sharing.

Benefits/merits	Risks/demerits
“Maximize the knowledge gained from the efforts and sacrifices of clinical trial participants” (ICMJE)	Privacy risk of participants unless data to be shared would be “de-identified” participant data
“Strengthening the science that is the foundation of safe and effective clinical care and public health practice” (CIOMS)	Risk to researcher/sponsor of impact of re-analysis on their original finding or commercial interests
Possibility of independent re-analysis of clinical trial results, including systematic review as well as subgroup analysis for personalized medicine	Risk to public health - impact of unfair/invalid secondary analysis
Increase the transparency and credibility of clinical trials	Burden of researchers to prepare their data/material obtained in their research in format possible to be shared with others

Summarized from the statements of the organizations cited in this manuscript.

There is another point to discuss concerning study registration requirement. A requirement of clinical trial outline information registration in a public database was stated by the ICMJE in 2004 (De Angelis et al., 2004) as a precondition for acceptance of a manuscript for publication of clinical trial results. This requirement was included in the DoH in 2008, and in the 2013 revision, the scope of the studies with a registration requirement was expanded from “clinical trial” to “every research study involving human subjects”. However, earlier in this century, not only trial outline registration at initiation, but also result registration at completion in a public database, has become a regulatory requirement in the United States (US) (FDA Act, 2007; NIH DHHS Final Rule, 2016), the European Union (EU) (EU Regulation 2014), Japan (Clinical Trial Act, 2017; MHLW, 2017) and other countries. A substantial lack of compliance with these regulations has been reported (Goldacre et al., 2018; The Lancet Oncology, 2019; DeVito et al., 2020; Piller, 2020). The paragraph 36 of the DoH requires result publication, but this paragraph does not refer explicitly to result uploading in public database, thus, this paragraph is generally understood as a journal publication requirement. Given that journal peer review takes time, thus study results often fail to be disclosed in timely manner. Additionally, not all journals provide open access and therefore restrict information transparency. On the other hand, result registration in public registries could be enforced by regulatory authorities, and be accessible in a timely manner to general public. The public disclosure practices compliance should be underlined as an “ethical obligation” in the DoH. The DoH should explicitly define it as a knowledge-sharing obligation concordance to the Declaration of Geneva (WMA Declaration of Geneva, 1948).

For the reasons as stated above, the DoH should include two additional requirements of study registration in a public database of 1) the data sharing plan at the initiation of and 2) full disclosure of results at the completion of a clinical trial.

## 5 DoT as an Ethical Basis of the European Union’s General Data Protection Regulation (GDPR)

Besides the well-recognized benefits of IPD sharing, one of the most heavily discussed risks of individual data sharing is privacy risk. Especially after the implementation of the EU’s General Data Protection Regulation (GDPR) (GDPR, 2016), the legal basis of secondary use of clinical trial data have been just under discussion between the European Commission (European Commission, 2019) and the European Data Protection Board (EDPB) (EDPB, 2018; EDPB, 2019). This regulation specific to the EU has great impact to the world because sharing personal data of the individual from the EU with countries outside the EU is governed by this regulation. Anonymized (de-identified) data is out of the scope of GDPR. ICMJE’s statement on IPD sharing is about this type of data, and anonymization methodology has been standardized by some clinical trial-related initiatives (PhUSE, 2015) or by each academic society. However, genuine anonymized data may have more limitations, and processing personal data into anonymized ones before secondary use also requires a legal framework. Justification of secondary use of personal data, pseudo-anonymized and/or coded, is a prerequisite.

In terms of GDPR, there are two possible avenues for justification: a) application of its article 89 of the GDPR for scientific research allows waiver of explicit consent of an individual, subject to appropriate privacy protection; b) justifiable consent to secondary use in line with Recital 33 (GDPR Recital 33) of the GDPR, which can be interpreted as broad consent, being subject to “recognized ethical standards”.

To provide justification to above mentioned both approaches, the WGE argues the combined use of DoH and DoT should be the ethical basis in the framework of GDPR for secondary use of IPD based in the following reasons:

1. The EDPB already recognized the DoH as the ethical foundation of informed consent (EDPB, 2018), thus the DoT should be the foundation of valid consent for future secondary use of personal data;
2. The EU Clinial Trial Regulation already defined such consent for secondary use separate from consent to clinical trial participation (EU Clinical Trial Regulation, 2014); and
3. The Council for International Organizations of Medical Sciences (CIOMS) have already recognized “Broad informed consent” to secondary use in its guidelines (CIOMS, 2016).

## 6 Conclusion

Based on the above described analysis, the WGE proposes revisions of the DoH necessary to facilitate expanding data-driven clinical science while assuring continued protection of research participants as follows:

1. The relationship between the DoH and DoT should be clearly described in the DoH. It should be clarified that not only intentional development of health-databases or biobanks, but any research activity must adhere to the DoT, where there is any possibility of secondary use or sharing with others of the data/material collected in the research.
2. Any future plan of sharing of data and/or material obtained in the research should be clearly described in a study protocol and ICF to be assessed by an ethics committee and to enable the candidate participants to make decision whether to accept this secondary use. This consent should be separately and independently obtained from the consent to participate in the proposed research, without impact on possible participation in the primary research.
3. The right of an individual to decide whether he/she wants to be informed of IFs should be assured.
4. In addition to the study registration requirement, registration requirements of “data sharing plan” and “study results” in publicly available databases should be explicitly defined as critical elements of physicians’ obligation of knowledge sharing.

The WGE believes this revision of the DoH to clarify linkage to the DoT will provide a solution for critical challenges of future-oriented research ethics.

## Data Availability

The original contributions presented in the study are included in the article/Supplementary Material, further inquiries can be directed to the corresponding author/s.
