# Corrigendum: Linking the Declarations of Helsinki and of Taipei: Critical Challenges of Future-Oriented Research Ethics

**DOI:** 10.3389/fphar.2020.637775

**Published:** 2021-02-08

**Authors:** Chieko Kurihara, Varvara Baroutsou, Sander Becker, Johan Brun, Brigitte Franke-Bray, Roberto Carlesi, Anthony Chan, Luis Francisco Collia, Peter Kleist, Luís Filipe Laranjeira, Kotone Matsuyama, Shehla Naseem, Johanna Schenk, Honorio Silva, Sandor Kerpel-Fronius, on behalf of Working Group on Ethics of the International Federation of Associations of Pharmaceutical Physicians and Pharmaceutical Medicine

**Affiliations:** ^1^ National Institutes for Quantum and Radiological Science and Technology, Chiba, Japan; ^2^ Independent Medical Consultant, and Consultant, Pharmaceutical Medicine, Athens, Greece; ^3^ Consultants in Pharmaceutical Medicine, Dover Heights, Australia; ^4^ LIF, Stockholm, Sweden; ^5^ Independent Consultant, Basel, Switzerland; ^6^ Independent Researcher, Bellagio, Italy; ^7^ Pfizer Biopharmaceuticals Group, Dublin, Ireland; ^8^ Craveri Pharma, Buenos Aires, Argentina; ^9^ Cantonal Ethics Committee, Zurich, Switzerland; ^10^ Eli Lilly & Co., Lisbon, Portugal; ^11^ Nippon Medical School, Tokyo, Japan; ^12^ Ferozsons Laboratories Ltd, Karachi, Pakistan; ^13^ PPH Plus GmbH & Co. KG, Hochheim am Main, Germany; ^14^ IFAPP Academy, New York, NY, United States; ^15^ Semmelweis University, Budapest, Hunbary

**Keywords:** research ethics, data science, medicines development, privacy protection, data sharing, declaration of Helsinki, declaration of Taipei

In the original article, there were errors.

In the **abstract**, “(Declaration of Taipei)” was deleted; in the **1 Introduction** section, “1948, in its second year” was changed to “in its second and third years”; in the **4 Individual Participant Data (IPD) Sharing and Trial Registration Requirement section**, parenthesis before and after “Supplementary Material”.

The authors apologize for this error and state that this does not change the scientific conclusions of the article in any way. The original article has been updated.

