# Supplementary material for: Linking the Declarations of Helsinki and of Taipei: Critical Challenges of Future-Oriented Research Ethics
Source: Front Pharmacol. 2020 Oct 29;11:579714. doi: 10.3389/fphar.2020.579714 (PMC7723451; doi:10.3389/fphar.2020.579714)
Supplement: Supplementary file 1 [file table1.docx]

Supplementary Material

**Selected key organizations with stated data sharing policy**

| 2003^[[1]](#endnote-1)^ | **United States National Institutes of Health (NIH)**  Data sharing requirement for research receiving its funding of more than 500,000 USD (Not limited to clinical trials) |
| --- | --- |
| 2011^[[2]](#endnote-2)^ | **The Wellcome Trust**  **The United Kingdom Medical Research Council**  **The World Bank**  **NIH and others**  Joint statement on data sharing requirement for research receiving their funding (Not limited to clinical trials) |
| 2012^[[3]](#endnote-3)^, | **United Kingdom Royal Society**  Statement on data sharing (Not limited to clinical trials) |
| 2013 ^[[4]](#endnote-4)^ | **European Federation of Pharmaceutical Industries and Associations (EFPIA)**  **Pharmaceutical Research and Manufacturers of America (PhRMA)**  Joint principles on responsible clinical trial data sharing |
| 2014^[[5]](#endnote-5)^^[[6]](#endnote-6)^ | **European Medicines Agency (EMA)**  Policy on publication of clinical trial results and submission of individual participant data (IPD) to be considered for sharing, as a requirement of clinical trial submitted to EMA (Policy 0070, EMA/240810/2013, superseded by EMA/144064/2019) |
| 2015^[[7]](#endnote-7)^ | **United States National Academy of Science, Institute of Medicine (IOM)**  Report on risks and benefit of clinical trial data sharing |
| 2015^[[8]](#endnote-8)^ | **Pharmaceutical Users Software Exchange (PhUSE)**  **Clinical Data Interchange Standards Consortium (CDISC)**  Standardization of anonymization for clinical trial IPD sharing |
| 2016^[[9]](#endnote-9)^ | **United States Department of Health and Human Services (DHHS)**  DHHS requires FDA-regulated clinical trials result information submission and does not require IPD sharing but recognizes value of IPD sharing and suggests possibility to register IPD in Clinica.Trial.gov. |
| 2016^[[10]](#endnote-10)^ | **Council for International Organizations of Medical Sciences (CIOMS)**  Recommendation in ethical guideline to facilitate data sharing of research involving human subjects |
| 2017^[[11]](#endnote-11)^ | **Japan Pharmaceutical Manufacturers Association (JPMA)**  Analysis on clinical trial data sharing including critical challenges for implementation |
| 2017^[[12]](#endnote-12)^ | **International Committee of Medical Journal Editors (ICMJE)**  Statement to require registration of IPD sharing plan for manuscript of clinical trial starting on or after January 2019 |
| 2017^[[13]](#endnote-13)^^[[14]](#endnote-14)^ | **Japanese Ministry of Health, Labor and Welfare**  Data sharing plan is included in the study completion report form under the Clinical Trial Act; A notification for clinical trials under the GCP Ordinance on clinical trial registration requirements includes data sharing plan |
| 2018^[[15]](#endnote-15)^ | **World Health Organization (WHO)**  International standards of management and data set of clinical trial registries. “Data sharing plan” is one of the required data items. |
| 2018^[[16]](#endnote-16)^ | **International Federation of Pharmaceutical Manufacturers & Associations (IFPMA)**  Joint principles to facilitate clinical trial IPD sharing |

**References**

1. National Institutes of Health (2003). *NIH data sharing policy and implementation guidance.* Available online at: <https://grants.nih.gov/grants/policy/data_sharing/data_sharing_guidance.htm> (accessed 20 June 2020). [↑](#endnote-ref-1)
2. Wellcome Trust. *Sharing research data to improve public health: full joint statement by funders of health research*. Available online at: <https://wellcome.ac.uk/what-we-do/our-work/sharing-research-data-improve-public-health-full-joint-statement-funders-health> (accessed 20 June 2020). [↑](#endnote-ref-2)
3. Royal Society (2012). *Science as an open enterprise: open data for open science*. Available online at: <https://royalsociety.org/~/media/policy/projects/sape/2012-06-20-saoe.pdf> (accessed 20 June 2020). [↑](#endnote-ref-3)
4. PhRMA, EFPIA (2013). *Principles for responsible clinical trial data sharing: our commitment to patients and researchers.* Available online at: <https://www.efpia.eu/media/25189/principles-for-responsible-clinical-trial-data-sharing.pdf> (accessed 20 June 2020). [↑](#endnote-ref-4)
5. European Medicines Agency (2014). *European Medicines Agency policy on publication of clinical data for medicinal products for human use*. EMA/240810/2013; 2 October 2014 [↑](#endnote-ref-5)
6. European Medicines Agency (2019). *European Medicines Agency policy on publication of clinical data for medicinal products for human use.* EMA/144064/2019. Available online at: https://www.ema.europa.eu/en/documents/other/european-medicines-agency-policy-publication-clinical-data-medicinal-products-human-use_en.pdf (accessed 20 June 2020). [↑](#endnote-ref-6)
7. Institute of Medicine (2015). Sharing clinical trial data: maximizing benefits, minimizing risk. (Washington, D.C.: National Academies Press). Available online at: <https://www.nap.edu/catalog/18998/sharing-clinical-trial-data-maximizing-benefits-minimizing-risk> (accessed 20 June 2020). [↑](#endnote-ref-7)
8. Pharmaceutical Users Software Exchange (2015). *PhUSE De-identification standards for CDISC SDTM 3.2. version: 1.01*. Available online at: <http://www.phuse.eu/data-transparency> (accessed 20 June 2020). [↑](#endnote-ref-8)
9. National Institutes of Health, Department of Health and Human Services．Clinical trials registration and results information submission. Final rule (2016)．*Federal Register*．2016 Sep 21; 81(183): 64981-5157． [↑](#endnote-ref-9)
10. Council for International Organizations of Medical Sciences (2016). *International ethical guidelines for health-related research involving humans*. Available online at: https://cioms.ch/wp-content/uploads/2017/01/WEB-CIOMS-EthicalGuidelines.pdf (accessed 20 June 2020). [↑](#endnote-ref-10)
11. Data Science Committee, Japan Pharmaceutical Manufacturers Association (2017). *Sharing of data of individual participant of clinical trial: CTDS (Clinical Trial Data Sharing)*. Japanese. Available online at: http://www.jpma.or.jp/medicine/shinyaku/tiken/allotment/pdf/ctds.pdf (accessed 20 June 2020). [↑](#endnote-ref-11)
12. Taichman DB, Sahni P, Pinborg A, Peiperl L, Laine C, James A, et al (2017)．Data sharing statements for clinical trials: A requirement of the International Committee of Medical Journal Editors. *PLoS Med*. 14, e1002315． [↑](#endnote-ref-12)
13. Clinical Trial Act. Act No. 16 of April 14, 2017. Available online at: https://www.mhlw.go.jp/file/06-Seisakujouhou-10800000-Iseikyoku/0000213334.pdf (accessed 20 June 2020). [↑](#endnote-ref-13)
14. Pharmaceutical Evaluation Division, Pharmaceutical Safety and Environmental Health Bureau, Ministry of Health, Labour and Welfare (2017). *On the clinical trial registration.* Yakuseiyakushinhatsu0326 No. 3. 2017 March 26. Japanese. Available online at: https://www.pmda.go.jp/files/000223575.pdf (accessed 20 June 2020). [↑](#endnote-ref-14)
15. World Health Organization (2018). *International Standards for Clinical Trial Registries. Ver. 3.0.* Available online at: https://www.who.int/publications-detail/international-standards-for-clinical-trial-registers (accessed 20 June 2020).. [↑](#endnote-ref-15)
16. International Federation of Pharmaceutical Manufacturers & Associations (2018). *IFPMA principles for responsible clinical trial data sharing*. Available online at: <https://www.ifpma.org/wp-content/uploads/2010/11/IFPMA-Principles_Data-Sharing-FINAL-w-QA-vF.pdf> (accessed 20 June 2020). [↑](#endnote-ref-16)
